# Postoperative Atrial Fibrillation in Patients Undergoing Cardiac Surgery

**DOI:** 10.7759/cureus.86428

**Published:** 2025-06-20

**Authors:** Marina Katerini, Ioanna V Papathanasiou, Lambrini Kourkouta, Konstantinos Koukourikos, Theodosios Paralikas, Maria Malliarou, Areti Tsaloglidou

**Affiliations:** 1 Cardiology, General Hospital of Pella - Hospital Unit of Edessa, Thessaloniki, GRC; 2 Nursing, University of Thessaly, Larissa, GRC; 3 Nursing, International Hellenic University, Thessaloniki, GRC

**Keywords:** arrythmias, atrial fibrillation, cardiac surgery, postoperative period, risk factors

## Abstract

Introduction

Atrial fibrillation (AF) is the most common arrhythmia occurring after cardiac surgery. It can lead to hemodynamic instability and significantly increase the risk of thromboembolic events.

Aim

The primary aim of this study was to assess the incidence of AF in patients undergoing cardiac surgery during the first postoperative week. The secondary aim was to investigate the influence of preoperative, intraoperative, and postoperative factors on the development of postoperative AF (POAF).

Materials and methods

This study included 140 patients of all ages and both sexes who underwent cardiac surgery and were admitted to the ICU of the General Hospital of Athens “Ippokrateio.” All participants had no prior history of AF but developed a POAF episode lasting more than 30 seconds within the first postoperative week. Data were collected from the patients’ medical records.

Results

Among the 140 patients, 42.9% developed POAF. The incidence of POAF was significantly higher in patients who were taking diuretics (62.2% vs. 33.7%; p = 0.001) and in those who underwent mitral valve replacement (64.3% vs. 37.5%; p = 0.010). POAF was also associated with lower ejection fraction (p = 0.003), higher preoperative CRP levels (p < 0.001), longer duration of intubation (p = 0.015), longer cross-clamp time (p = 0.016), and extended ICU stay (p = 0.005). Furthermore, postoperative hemoglobin (p = 0.050) and hematocrit (p = 0.044) levels were significantly lower in POAF cases, while postoperative CRP levels were significantly higher (p < 0.001).

Conclusions

POAF is associated with multiple risk factors, including mitral valve surgery, prolonged ICU stay, low ejection fraction, extended cross-clamp time, and longer ventilation duration. Additionally, the use of diuretics and elevated CRP levels were significantly linked to the occurrence of POAF.

## Introduction

Increased life expectancy and the rising prevalence of cardiovascular disease in older populations have led to a significant increase in cardiac surgeries. As people live longer, age-related heart conditions requiring surgical intervention are becoming more common [1]. Postoperative atrial fibrillation (POAF) is the most frequent sustained arrhythmia following cardiac surgery and is associated with longer hospital stays, increased healthcare costs, complications, thromboembolic events, and higher mortality rates [2].

Overall, approximately 15-30% of cardiac surgery patients develop POAF [3]. The incidence varies by type of surgery: patients undergoing coronary artery bypass grafting (CABG) have a relatively lower risk (about 20%), whereas those undergoing valve replacement surgeries have a higher incidence (around 50%). The risk is even greater in patients undergoing combined CABG and valve procedures [4]. Rates of POAF also remain high following aortic surgery, ranging between 30% and 50% [5].

POAF is defined as new-onset atrial fibrillation (AF) occurring after surgery in patients without a prior history of arrhythmia [6]. The risk factors associated with the development of POAF can be broadly categorized into patient-related factors, such as age, sex, and comorbidities, and surgery-related factors, including the type of surgery performed. Additionally, there are reversible causes of POAF, the correction of which can potentially restore sinus rhythm promptly [7].

The aim of the present study is to evaluate the incidence of AF in patients undergoing cardiac surgery during the first postoperative week. The secondary aim is to examine the influence of preoperative, intraoperative, and postoperative variables on the development of POAF.

The pathogenesis of POAF is multifactorial and differs from that of nonsurgical AF, as cardiac surgery introduces specific conditions that contribute to its onset [8]. Key mechanisms involved in POAF include atrial myocardial injury, pericardial effusion and inflammation, gap junction uncoupling, peri-atrial fat metabolism, autonomic nervous system modulation, ion channel alterations, re-entry circuits, and ectopic activity in the pulmonary veins [9].

Beyond advanced age, other clinical risk factors for POAF after cardiac surgery include increased left atrial diameter, reduced left ventricular ejection fraction, and a history of heart failure (HF), chronic obstructive pulmonary disease, hypertension, and myocardial infarction. However, a meta-analysis found that body mass index, beta-blocker use, sex, and dyslipidemia were not significantly associated with POAF incidence [10].

Advanced age is not only a risk factor for morbidity and mortality after cardiac surgery [1], but also one of the strongest predictors of POAF, with incidence rates rising sharply after age 50 [11]. For example, a study by Shen et al. reported that the incidence of POAF was 25% at age 60, 40% at age 70, and 50% at age 80 [12].

Management of POAF includes the use of beta-blockers, amiodarone, statins, and oral anticoagulants. Moreover, several studies have demonstrated that postoperative supplementation with magnesium [13] and vitamin D [14] can significantly reduce the risk of developing POAF.

## Materials and methods

Study design and data collection

This study utilized a retrospective chart review to collect data from existing medical records and telemetry/ECG monitoring documentation, as outlined below. Information gathered included demographic and social characteristics, medical history, type of surgery, medications, and the results of relevant laboratory and imaging tests.

The study sample comprised 140 patients, both men and women of all ages, who underwent cardiac surgery and were admitted to the ICU of the General Hospital of Athens “Ippokrateio.” Patient data were collected over a 22-month period, from September 2020 to June 2022.

Inclusion criteria required that patients have no prior history of AF and experience a POAF episode lasting more than 30 seconds during the first week of hospitalization. All patients had continuous heart rhythm and heart rate monitoring via ECG telemetry during their stay in the resuscitation unit, where they typically remained for the first 48 hours post-surgery. Patients were then transferred to the cardiac surgery ward, where telemetry monitoring was used if arrhythmia was suspected. The preferred method for accurate rhythm assessment was ECG or telemetry, both performed by a physician and monitored by a trained ward nurse.

Exclusion criteria

Patients were excluded from the study if they met any of the following criteria: undergoing emergency surgery, being under 18 years of age, having a history of AF (either paroxysmal or chronic), or presenting with hepatic dysfunction, uncontrolled HF, or a history of previous cardiac surgery (redo surgery).

Statistical analysis

Quantitative variables were presented as mean ± SD or as median with IQR, while categorical variables were expressed as absolute numbers and percentages. The chi-square test and Fisher’s exact test were used to compare proportions. For comparisons of continuous variables between patients who developed POAF and those who did not, Student’s t-test and the Mann-Whitney U test were applied, as appropriate.

A stepwise logistic regression analysis (entry criterion: p = 0.05; removal criterion: p = 0.10) was conducted to identify independent predictors of POAF. Adjusted ORs with 95% CIs were calculated from the regression model. All p-values were two-tailed, and statistical significance was set at p < 0.05. Analyses were performed using IBM SPSS Statistics for Windows, Version 27.0 (Released 2019; IBM Corp., Armonk, NY, USA).

Study ethics

The study protocol was reviewed and approved by the Scientific Council and the Research Ethics Committee of the General Hospital of Athens “IPPOKRATEIO,” granting access to electronic medical records and permission to collect and process data. The study was conducted in accordance with the ethical principles outlined in the Declaration of Helsinki.

## Results

The study sample included 140 patients who underwent cardiac surgery, the majority of whom were male (105 patients, 75%). In terms of age distribution, 33 patients (23.6%) were under 60 years old, 56 (40.0%) were between 61 and 70 years old, and 51 (36.4%) were aged 71 years or older. Regarding smoking status, 41 patients (29.3%) were active smokers, 48 (34.3%) were nonsmokers, and 51 (36.4%) were former smokers. Among the active smokers, 18 patients (43.9%) reported smoking one to 15 cigarettes per day, while 23 (56.1%) smoked more than 16 cigarettes daily. The demographic and clinical characteristics of the study population are summarized in Table 1.

**Table 1:** Demographic characteristics of the study population ¹ Refers only to active smokers BSA, body surface area

Characteristic	Category	n	%
Gender	Men	105	75
	Women	35	25
Age (years)	≤60	33	23.6
	61-70	56	40
	≥71	51	36.4
BMI	Normal	38	27.1
	Overweight	60	42.9
	Obese	42	30
Smoking status	Active smokers	41	29.3
	Nonsmokers	48	34.3
	Former smokers	51	36.4
Cigarettes/day¹	1-15	18	43.9
	≥16	23	56.1
Continuous variables		Mean (SD)	Median (IQR)
Age		67.3 (8.6)	68 (61-74)
BSA		1.93 (0.17)	1.92 (1.82-2.06)
BMI		28 (4.3)	27.7 (24.7-30.7)

The clinical characteristics of the study participants are summarized in Table 2. Among the 140 patients, 73 (52.1%) had coronary artery disease (CAD), 44 (31.4%) had valvular heart disease, and 23 (16.4%) had both CAD and valvular disease. A family history of CAD was reported by 52 patients (37.1%). Based on the New York Heart Association (NYHA) classification, 15 patients (10.7%) were categorized as NYHA I, 104 (74.3%) as NYHA II, and 21 (15.0%) as NYHA III.

**Table 2:** Clinical characteristics of study participants ¹ Multiple responses were allowed ACEi, angiotensin-converting enzyme inhibitors; AF, atrial fibrillation; ARBs, angiotensin II receptor blockers; AVR, aortic valve replacement; BSA, body surface area; CABG, coronary artery bypass grafting; CAD, coronary artery disease; CCBs, calcium channel blockers; CKD, chronic kidney disease; COPD, chronic obstructive pulmonary disease; CPK, creatine phosphokinase; CVA, cerebrovascular accident; DBP, diastolic blood pressure; DM, diabetes mellitus; ECC, extracorporeal circulation; EF, ejection fraction; HCT, hematocrit; HGB, hemoglobin; ICU, intensive care unit; K, potassium; LDH, lactate dehydrogenase; MVR, mitral valve replacement; NYHA, New York Heart Association; SBP, systolic blood pressure; SR, sinus rhythm

Clinical characteristics		n	%
Heart disease	CAD	73	52.1
	Heart valve disease	44	31.4
	CAD and heart valve disease	23	16.4
Family history	No	88	62.9
	Yes	52	37.1
ΝΥΗΑ	I	15	10.7
	II	104	74.3
	III	21	15
Comorbidities	Hypertension	132	94.3
	DM	53	37.9
	COPD	27	19.3
	Dyslipidemia	120	85.7
	CVA	14	10
	CKD	8	5.7
Medication	Aspirin	96	68.6
	Clopidogrel	40	28.6
	Beta-blockers	111	79.3
	ACEi	63	45
	ARBs	36	25.7
	CCBs	28	20
	Statins	116	82.9
	Diuretics	45	32.1
	Nitrates	31	22.1
	Inotropic drugs	62	44.3
Type of surgery¹	CABG	91	65
	AVR	39	27.9
	MVR	28	20
ECC	No	6	4.3
	Yes	134	95.7
ECG at discharge	SR	131	93.6
	AF	5	3.6
	Paced rhythm	4	2.9
In-hospital mortality	No	137	97.9
	Yes	3	2.1
Continuous variables		Mean (SD)	Median (IQR)
SBP		144.8 (8.1)	145 (142-151)
DBP		79 (4)	79 (77-82)
EF (%)		52.4 (9.6)	55 (47.5-60)
WBC (pre)		8.1 (2.2)	7.7 (6.6-9.1)
HGb (pre)		13.5 (1.4)	13.9 (12.6-14.5)
HCT (pre)		41.3 (4.7)	42.2 (39.2-44.2)
Creatinine (pre)		0.99 (0.24)	0.9 (0.9-1.1)
CRP (pre)		1.52 (1.48)	0.9 (0.4-2.2)
K (pre)		4.55 (0.37)	4.5 (4.3-4.8)
LDH (pre)		324.3 (512.8)	256 (227.5-311.5)
CPK (pre)		92.6 (84.3)	76 (51-97)
ECC (min)		97.6 (38.2)	97 (76.5-118.5)
Duration of operation (hours)		4.32 (0.92)	4 (4-5)
Ventilation support (hours of intubation)		19.7 (12.8)	17 (11-24)
Cross-clamping time (minutes)		65.2 (38.3)	66.5 (46-91.5)
ICU stay (days)		2.8 (1.28)	3 (2-4)
WBC (post)		10.8 (3)	10.7 (8.6-12.7)
HGB (post)		11 (2.8)	10.9 (9.8-11.9)
HCT (post)		33 (4.9)	33.5 (29.4-36.2)
Creatinine (post)		1.08 (0.32)	1 (0.9-1.2)
K (post)		4.21 (0.28)	4.1 (4-4.4)
LDH (post)		459.5 (287.8)	436 (348.5-505)
CPK (post)		425.8 (215)	348 (305.5-505.5)
CRP (post)		1.9 (1.6)	1.2 (0.7-2.8)

Hypertension was the most prevalent comorbidity, observed in 132 patients (94.3%), followed by dyslipidemia in 120 (85.7%), diabetes mellitus in 53 (37.9%), chronic respiratory failure in 27 (19.3%), stroke in 14 (10.0%), and chronic kidney disease in eight patients (5.7%). Regarding medication use, 116 patients (82.9%) were on statins, 111 (79.3%) were receiving β-blockers, and 96 (68.6%) were taking aspirin.

In terms of surgical procedures, 91 patients (65.0%) underwent CABG, 39 (27.9%) had aortic valve replacement (AVR), and 28 (20.0%) underwent mitral valve replacement (MVR). The in-hospital mortality rate among the study population was 2.1% (three patients).

Postoperative arrhythmia was observed in 66 patients (47.1%). Among these, 60 patients (42.9%) experienced episodes of POAF, while six patients (4.2%) developed third-degree atrioventricular block during hospitalization. The diagnosis of in-hospital POAF was confirmed in all cases using a 12-lead ECG. This diagnostic test was performed either following the detection of arrhythmia through continuous ECG monitoring, particularly during the first 48 hours after cardiac surgery, or during routine physical examination.

**Figure 1:**
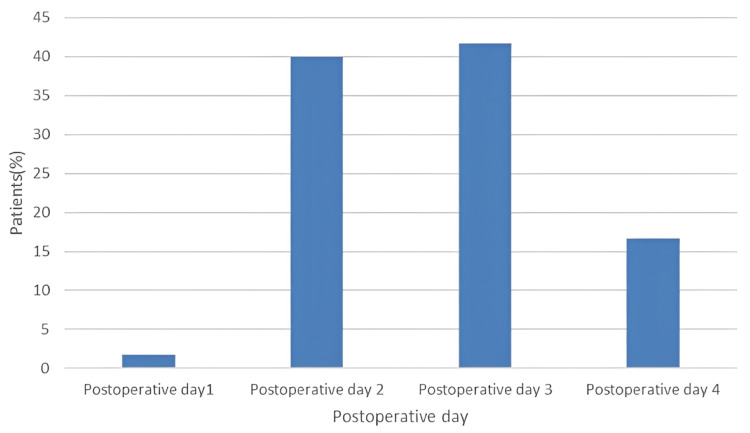
Frequency of AF occurrence during the first four postoperative days AF, atrial fibrillation

All cases of POAF were treated with amiodarone. Notably, every POAF episode occurred within the first four days following surgery, with the highest incidence observed on postoperative day 2 (40.0%) and day 3 (41.7%). Figure 1 illustrates the frequency of POAF across the first four postoperative days.

The association between demographic characteristics and POAF is presented in Table 3. The incidence of POAF was significantly higher in women than in men (57.1% vs. 38.1%; p = 0.049). No significant associations were found between POAF and other demographic variables.

**Table 3:** Association between demographic characteristics and POAF ^+^ p-value from Pearson’s chi-square test ^‡^ p-value from Student’s t-test ¹ Refers only to active smokers BSA, body surface area; POAF, postoperative atrial fibrillation

Demographic characteristics		POAF		χ² (df)	p
		No (n = 80; 57.1%)	Yes (n = 60; 42.9%)		
		n (%)	n (%)		
Gender	Men	65 (61.9)	40 (38.1)	3.89 (1)	0.049^+^
	Women	15 (42.9)	20 (57.1)		
Age	≤60	20 (60.6)	13 (39.4)	1.24 (2)	0.537^+^
	61-70	34 (60.7)	22 (39.3)		
	71+	26 (51.0)	25 (49.0)		
BMI	Normal	22 (57.9)	16 (42.1)	0.14 (2)	0.932^+^
	Overweight	35 (58.3)	25 (41.7)		
	Obese	23 (54.8)	19 (45.2)		
Smoking	Active smokers	26 (63.4)	15 (36.6)	4.77 (2)	0.092^+^
	Nonsmokers	31 (64.6)	17 (35.4)		
	Former smokers	23 (45.1)	28 (54.9)		
Cigarettes/day¹	1-15	14 (77.8)	4 (22.2)	2.85 (1)	0.091^+^
	16+	12 (52.2)	11 (47.8)		
Continuous variables		Mean (SD)	Mean (SD)	t (df)	
Age		66.5 (8.6)	68.3 (8.6)	1.22 (138)	0.225^‡^
BSA		1.94 (0.17)	1.93 (0.18)	-0.42 (138)	0.674^‡^
BMI		28.2 (4.6)	27.7 (3.8)	-0.62 (138)	0.538^‡^

The association between clinical characteristics and the occurrence of POAF is presented in Table 4. The incidence of POAF was significantly higher among patients who were receiving diuretics and those who underwent MVR. Additionally, POAF was significantly associated with lower ejection fraction, elevated preoperative CRP levels, longer intubation time, extended cross-clamp duration, and prolonged ICU stay.

**Table 4:** Association between clinical characteristics and POAF ^+^ p-value from Pearson’s chi-square test ^++^ p-value from Fisher’s exact test ^‡^ p-value from Student’s t-test ^‡‡^ p-value from Mann-Whitney test ¹ Multiple responses were allowed ACEi, angiotensin-converting enzyme inhibitors; ARBs, angiotensin II receptor blockers; AVR, aortic valve replacement; CABG, coronary artery bypass surgery; CAD, coronary artery disease; CCBs, calcium channel blockers; CKD, chronic kidney disease; COPD, chronic obstructive pulmonary disease; CPK, creatine phosphokinase; CVA, cerebral vascular accident; DBP, diastolic blood pressure; DM, diabetes mellitus; ECC, extracorporeal circulation; EF, ejection fraction; HCT, hematocrit; HGB, hemoglobin; LDH, lactate dehydrogenase; MVR, mitral valve replacement; NYHA, New York Heart Association; SBP, systolic blood pressure

Clinical characteristics		POAF		χ² (df)	p
		No (n = 80; 57.1%)	Yes (n = 60; 42.9%)		
		n (%)	n (%)		
Heart disease	CAD	46 (63.0)	27 (37.0)	2.62 (2)	0.269^+^
	Heart valve disease	21 (47.7)	23 (52.3)		
	CAD and heart valve disease	13 (56.5)	10 (43.5)		
Family history	No	53 (60.2)	35 (39.8)	0.92 (1)	0.337^+^
	Yes	27 (51.9)	25 (48.1)		
ΝΥΗΑ	I	10 (66.7)	5 (33.3)	1.35 (2)	0.510^+^
	II	60 (57.7)	44 (42.3)		
	III	10 (47.6)	11 (52.4)		
Comorbidities					
Hypertension	No	7 (87.5)	1 (12.5)	3.19 (1)	0.138^++^
	Yes	73 (55.3)	59 (44.7)		
DM	No	52 (59.8)	35 (40.2)	0.65 (1)	0.421^+^
	Yes	28 (52.8)	25 (47.2)		
COPD	No	67 (59.3)	46 (40.7)	1.11 (1)	0.293^+^
	Yes	13 (48.1)	14 (51.9)		
Dyslipidemia	No	11 (55.0)	9 (45.0)	0.04 (1)	0.834^+^
	Yes	69 (57.5)	51 (42.5)		
CVA	No	71 (56.3)	55 (43.7)	0.32 (1)	0.569^+^
	Yes	9 (64.3)	5 (35.7)		
CKD	No	76 (57.6)	56 (42.4)	0.18 (1)	0.724^++^
	Yes	4 (50.0)	4 (50.0)		
Medication					
Aspirin	No	28 (63.6)	16 (36.4)	1.11 (1)	0.293^+^
	Yes	52 (54.2)	44 (45.8)		
Clopidogrel	No	61 (61.0)	39 (39.0)	2.13 (1)	0.145^+^
	Yes	19 (47.5)	21 (52.5)		
Beta-blockers	No	16 (55.2)	13 (44.8)	0.06 (1)	0.810^+^
	Yes	64 (57.7)	47 (42.3)		
ACEi	No	42 (54.5)	35 (45.5)	0.47 (1)	0.492^+^
	Yes	38 (60.3)	25 (39.7)		
ARB	No	63 (60.6)	41 (39.4)	1.95 (1)	0.163^+^
	Yes	17 (47.2)	19 (52.8)		
CCB	No	67 (59.8)	45 (40.2)	1.64 (1)	0.200^+^
	Yes	13 (46.4)	15 (53.6)		
Statins	No	13 (54.2)	11 (45.8)	0.11 (1)	0.746^+^
	Yes	67 (57.8)	49 (42.2)		
Diuretics	No	63 (66.3)	32 (33.7)	10.16 (1)	0.001^+^
	Yes	17 (37.8)	28 (62.2)		
Nitrates	No	63 (57.8)	46 (42.2)	0.09 (1)	0.769^+^
	Yes	17 (54.8)	14 (45.2)		
Amiodarone	No	80 (57.6)	59 (42.4)	1.34 (1)	0.429^++^
	Yes	0 (0.0)	1 (100.0)		
Inotropic drugs	No	44 (56.4)	34 (43.6)	0.04 (1)	0.844^+^
	Yes	36 (58.1)	26 (41.9)		
Type of surgery¹					
CABG	No	25 (51.0)	24 (49.0)	1.15 (1)	0.283^+^
	Yes	55 (60.4)	36 (39.6)		
AVR	No	57 (56.4)	44 (43.6)	0.07 (1)	0.786^+^
	Yes	23 (59.0)	16 (41.0)		
MVR	No	70 (62.5)	42 (37.5)	6.56 (1)	0.010^+^
	Yes	10 (35.7)	18 (64.3)		
ECC	No	5 (83.3)	1 (16.7)	1.76 (1)	0.238^++^
	Yes	75 (56.0)	59 (44.0)		
In-hospital mortality	No	80 (58.4)	57 (41.6)	4.09 (1)	0.076^++^
	Yes	0 (0.0)	3 (100.0)		
Continuous variables		Mean (SD)	Mean (SD)	t (df)	
SBP		144.5 (8.8)	145.3 (7.2)	0.56 (138)	0.579^‡^
DBP		79.1 (4.1)	78.9 (3.8)	-0.33 (138)	0.746^‡^
Continuous variables		Median (IQR)	Median (IQR)	U value	
EF (%)		55 (50-60)	50 (45-55)	1702	0.003^‡‡^
WBC (pre)		7.8 (6.5-9.1)	7.7 (6.6-9.1)	2322	0.743^‡‡^
HGb (pre)		14.1 (12.8-14.5)	13.4 (12.3-14.5)	2027	0.116^‡‡^
HCT (pre)		42.5 (39.4-43.9)	42 (38.5-44.5)	2212	0.429^‡‡^
Creatinine (pre)		0.9 (0.9-1)	0.95 (0.8-1.1)	2187.5	0.357^‡‡^
CRP (pre)		0.5 (0.2-0.85)	2.25 (1.55-3.5)	388	<0.001^‡‡^
K (pre)		4.5 (4.3-4.8)	4.5 (4.2-4.8)	2062.5	0.150^‡‡^
LDH (pre)		247.5 (223-304)	272.5 (235.5-321)	1972.5	0.072^‡‡^
CPK (pre)		79.5 (62.5-99.5)	75 (45-95)	2052	0.143^‡‡^
ECC (min)		91 (76.5-113.5)	102.5 (76.5-125)	2059.5	0.152^‡‡^
Duration of operation (hours)		4 (4-5)	4 (4-5)	2068.5	0.135^‡‡^
Ventilation support (hours of intubation)		14 (10.5-20)	20 (12-26)	1824.5	0.015^‡‡^
Cross-clamping time (minutes)		61 (44-84)	73.5 (55.5-99)	1826.5	0.016^‡‡^
ICU stay (days)		2 (2-3)	3 (2-4)	1756	0.005^‡‡^
WBC (post)		10.5 (8.4-12.6)	11.1 (8.7-12.9)	2243.5	0.510^‡‡^
HGB (post)		11.3 (10.2-12)	10.4 (9.4-11.4)	1935	0.050^‡‡^
HCT (post)		34.3 (30.5-36.5)	32.1 (28.3-35.7)	1922	0.044^‡‡^
Creatinine (post)		1 (0.9-1.15)	1.1 (0.9-1.3)	1951	0.055^‡‡^
K (post)		4.15 (4-4.4)	4.1 (4-4.4)	2386	0.953^‡‡^
LDH (post)		427.5 (334.5-492.5)	449.5 (370-512)	2173.5	0.340^‡‡^
CPK (post)		386 (305.5-535.5)	346.5 (304-469.5)	2218.5	0.445^‡‡^
CRP (post)		0.8 (0.4-1.1)	2.8 (2.1-3.9)	306	<0.001^‡‡^

The results of the multiple logistic regression analysis are presented in Table 5. Two factors were found to be significantly associated with the development of POAF: undergoing MVR and the length of ICU stay. Specifically, a longer ICU stay was associated with an increased likelihood of developing POAF. Additionally, patients who underwent MVR were 2.77 times more likely to experience POAF compared to those who did not.

**Table 5:** Multiple logistic regression results with POAF as the dependent variable MVR, mitral valve replacement; POAF, postoperative atrial fibrillation

Variable	OR	95% CI	p
ICU stay (days)	1.39	1.05-1.83	0.023
MVR (yes vs. no)	2.77	1.15-6.67	0.023

## Discussion

Despite advances in both prevention and treatment, POAF remains one of the most common and clinically significant arrhythmias following cardiac surgery. Its incidence remains high, ranging from 20% to 55% [15], and it is associated with a fourfold increase in thromboembolic events and a twofold increase in mortality compared to patients who do not develop POAF [16,17]. Additionally, affected patients typically experience a longer hospital course, with an average increase of up to two days in the ICU and up to three days in total hospital stay [18].

In the present study, the majority of POAF episodes occurred within the first four postoperative days, peaking on the second and third days. Similar findings were reported in the Afrodite study, a prospective multicenter cohort study evaluating AF recurrence after CABG, where arrhythmias most frequently occurred on the third and fourth postoperative days [19].

Advanced age is widely recognized as one of the strongest risk factors for the development of AF after open-heart surgery. A retrospective cohort study reported that patients who developed POAF were significantly older (70.6 ± 10.7 years) compared to those who did not (60.4 ± 16.4 years, p = 0.001) [20]. In our study, patients with POAF were also older (68.3 ± 8.6 vs. 66.3 ± 8.8 years), although the difference did not reach statistical significance. However, a statistically significant association was observed in patients who underwent MVR (p = 0.010). This finding is consistent with a study by Dokko et al. (2022), which found a higher incidence of POAF following mitral valve surgery compared to other cardiac procedures [21]. Another study reported POAF rates ranging from 14% to 42% following mitral valve surgery, with approximately 23% of patients developing POAF after MVR and 15% after mitral valve repair [22].

A retrospective case-control study involving 388 CABG patients at Townsville University Hospital found that increased cross-clamping time was associated with POAF [23]. In the present study, longer cross-clamping time was also associated with POAF (p = 0.016), although the association was not statistically significant. In contrast, a recent single-center prospective study (2023) found no significant association between cross-clamping time and POAF incidence [24].

A retrospective study conducted at King Faisal Specialist Hospital and Research Centre in Jeddah, Saudi Arabia, showed that patients who developed POAF had a significantly lower ejection fraction (44.8%) compared to those without POAF (56.7%) (p = 0.001) [25]. The same study also reported that ICU stay, ventilation time, and total hospital stay were significantly longer in POAF patients. Similarly, our findings demonstrated statistically significant associations between POAF and lower ejection fraction (p < 0.012), prolonged mechanical ventilation (p = 0.008), and extended ICU stay (p < 0.001). Supporting this, a large multi-institutional study by Hawkins et al. in the southeastern United States, which included 27,307 cardiac surgery patients, also found an association between POAF and ICU length of stay [26]. Our multivariate analysis confirmed that patients who developed POAF were more likely to have undergone MVR and experienced longer ICU stays, with MVR associated with a 2.77-fold increased likelihood of developing POAF.

In our study, postoperative CRP levels were significantly elevated in patients who developed POAF (p < 0.001). This aligns with findings from a cross-sectional study of patients undergoing AVR, which reported significantly higher peak CRP levels in those who developed POAF [27]. These results support the hypothesis that inflammation plays a role in the development of POAF.

Renal function has also been implicated in POAF risk. A study by Limite et al. [28] found that a lower estimated glomerular filtration rate (eGFR) was associated with POAF in valve surgery patients. In our study, patients with elevated postoperative creatinine levels were more likely to develop POAF, although the difference was not statistically significant (p = 0.055).

Preoperative diuretic use has also been associated with POAF. A retrospective observational case-control study investigating predictors of new-onset POAF in off-pump CABG patients found that older age, higher baseline systolic blood pressure, preoperative diuretic use, blood transfusion, atrial dilation, and postoperative use of positive inotropic agents were all associated with increased risk of POAF [29].

Limitations

The main limitation of this study is the relatively small sample size. Additionally, data were collected from a single hospital, and diagnostic or monitoring practices, such as the use of electrocardiography or telemetry, may differ across institutions. Some brief or asymptomatic episodes of POAF may have gone undetected, particularly if they occurred outside of continuous monitoring periods.

Future implications

Further large-scale, multicenter studies are needed to improve the generalizability of findings and account for variations in clinical practice, including monitoring techniques and diagnostic protocols. Future research should also investigate the long-term impact of undiagnosed POAF on patient outcomes, with the aim of informing optimal screening and management strategies.

## Conclusions

This study identified a significant association between POAF and both longer ICU stays and MVR. In addition, lower ejection fraction, longer cross-clamp duration, and extended ventilation time were found to be important factors that increase the risk of POAF following cardiac surgery.
